# Aging of visual word perception is related to decreased segregation within and beyond the word network in the brain

**DOI:** 10.3389/fnagi.2024.1483449

**Published:** 2024-12-05

**Authors:** Licheng Xue, Tianying Qing, Yating Lv, Jing Zhao

**Affiliations:** ^1^School of Early Childhood Education, Hangzhou Polytechnic, Zhejiang, China; ^2^Helmholtz Institute, Utrecht University, Utrecht, Netherlands; ^3^Center for Cognition and Brain Disorders, The Affiliated Hospital of Hangzhou Normal University, Zhejiang, China; ^4^Zhejiang Key Laboratory for Research in Assessment of Cognitive Impairments, Zhejiang, China; ^5^Jing Hengyi School of Education, Hangzhou Normal University, Zhejiang, China; ^6^Zhejiang Philosophy and Social Science Laboratory for Research in Early Development and Childcare, Hangzhou Normal University, Zhejiang, China

**Keywords:** aging, visual word processing, functional connectivity, resting-state fMRI, neural dedifferentiation hypothesis

## Abstract

**Introduction:**

We investigated the neural correlates of cognitive decline in visual word perception from the perspective of intrinsic brain networks.

**Methods:**

A total of 19 healthy older adults and 22 young adults were recruited to participate in two functional magnetic resonance imaging (fMRI) sessions (one resting-state session and one for localizer tasks), along with a visual word perceptual processing task. We examined age-related alterations in resting-state functional connectivity (FC) within the word network, as well as between the word network and other networks. We tested their associations with behavioral performance in word and symbol-form processing.

**Results:**

We found that, compared to young adults, older adults exhibited increased FC between the two word-selective regions in the left and right ventral occipitotemporal cortex (vOT). Additionally, older adults exhibited increased FC between these two word-selective regions and non-word-selective regions. Notably, these FC alterations correlated with individual differences in behavioral performance in visual word perception.

**Discussion:**

These results suggest that cognitive decline in visual word perception is associated with decreased segregation within and beyond the word network in the aging brain. Our findings support the neural dedifferentiation hypothesis for cognitive decline in visual word processing and improve our understanding of interactive neural specialization theory.

## Introduction

Older adults experience various forms of perceptual decline as they age (Koen and Rugg, 2019). Given the rapid pace at which the population is aging, identifying the factors associated with perceptual decline in this demographic is crucial (Koen et al., 2020). Visual words represent a typical perceptual expert stimulus, yet many older adults struggle with processing them effectively (Salthouse, 2019; Xue et al., 2023). Thus, we further explored the neural correlates of perceptual decline in visual word processing in older adults from an intrinsic functional brain network perspective.

It has been widely observed that older adults experience a deterioration in the processing of visual words (Koen and Rugg, 2019; Salthouse, 2019; Xue et al., 2023). The majority of previous studies have focused on how language and reading skills change with age, demonstrating that older adults experience a decline in these processes (Gollan and Brown, 2006; Han and Liang, 2019; Su et al., 2017). For example, Su et al. (2017) compared the eye movements of older and young adults while reading sentences. The results revealed that older adults spend more time reading a sentence, indicating an aging effect on the visual encoding of multiple words during reading in Chinese (Su et al., 2017). However, relatively few studies have examined the changes in perceptual processing of visual words related to aging, although this process is fundamental to visual word recognition and reading (Ehri, 1995; Zhao et al., 2014). Recently, Xue et al. (2023) used a visual matching task and found that older adults performed worse than young adults in visual word perception. This finding suggests that older adults experience a perceptual decline in visual word processing.

Further, previous research explored the neural correlates of decline in visual word perceptual processing among older adults. According to the neural dedifferentiation theory, one hypothesis posits that the age-related decline in visual word processing is linked to reduced selectivity in neural responses to words (see Koen and Rugg, 2019 for a review). Consistent with this hypothesis, previous studies have shown that older adults exhibit decreased selectivity for words in the left ventral occipitotemporal cortex (vOT) compared to young adults (Park et al., 2004; Xue et al., 2023). The left vOT is known to show stronger neural responses to visual words compared to other visual stimuli, such as checkerboards, geometric symbols, and scrambled words, in young adults (e.g., Cohen et al., 2002; Cohen and Dehaene, 2004; Tarkiainen et al., 1999). Park et al. (2004) conducted a memory task to examine differences in neural response selectivity to word-like stimuli (pseudowords) in the left vOT between younger and older adults. They found that young adults exhibited stronger neural responses to pseudowords than to symbol strings, whereas older adults did not show significant differences between these two types of stimuli. Similarly, Xue et al. (2023) found reduced selectivity in neural responses to words in the left vOT among older adults, and decreased neural responses to words were correlated with a decline in visual word perceptual processing.

Visual word perception is a complex cognitive process that requires the coordination of multiple neural substrates and the involvement of specific brain regions (Beaulieu et al., 2005; Johnson, 2000, 2005; Xue et al., 2020). Moreover, spontaneous neural activity persists even when the brain is not engaged in specific tasks and is crucial for maintaining normal physiological functions (Xue et al., 2020). Therefore, to reveal the brain’s intrinsic functional architecture, it is essential to measure spontaneous neural activity during the resting state alongside task-evoked neural activity (Deco et al., 2011; Fox et al., 2005; van Diessen et al., 2015). The resting-state fMRI functional connectivity (FC) approach is commonly employed to explore the characteristics of brain networks involved in word processing (Hampson et al., 2006; He, 2015; Zhang et al., 2014). For instance, He (2015) found that skilled readers exhibited stronger FC between the left vOT region and several language-related brain areas, such as Broca’s area, compared to illiterate participants. At the individual level, FC between the left vOT and language-related brain areas was positively correlated with visual word reading performance (He, 2015; Zhang et al., 2014). Similarly, previous studies have shown that FC within the word brain network is stronger in adults with good reading ability compared to those with poor reading ability (Hampson et al., 2006). These findings suggest that resting-state FC reliably reflects the coordination of brain networks related to visual word processing. However, little is known about the resting-state brain network characteristics associated with declined word perception in older adults. From an intrinsic brain network perspective, it remains unclear whether neural dedifferentiation occurs in the cognitive decline in visual word processing in older adults.

Therefore, the present study aimed to investigate the neural correlates of aging on visual word perception from the perspective of functional brain networks. Specifically, we addressed two questions: (a) How does FC differ within and between the word network and other networks in older adults compared to young adults? and (b) How is the change in FC related to the decline in visual word perception? We utilized a block-designed word localizer run to identify word-selective brain regions (Park et al., 2004; Xue et al., 2023). We employed a content-irrelevant location judgment task to minimize the potential confounding effects related to task modulation. Following the fMRI experiment, we used a visual matching task to assess word perception in both young and older adults (Xue et al., 2023). To account for general aging effects, we included symbol pictures as a control condition. Based on previous findings (Hampson et al., 2006; He, 2015; Zhang et al., 2014), we hypothesized that (1) older adults and young adults would exhibit differences in FC within and between the word network and other networks. (2) changes in FC would be related to the individual’s performance in the visual word-matching task.

## Methods

### Participants

For this study, we recruited 19 healthy older adults (mean age = 64.74 ± 4.33 years; 11 men) and 22 young adults (mean age = 21.68 ± 1.76 years; 11 men). All participants were right-handed individuals with no history of encephalopathy or mental illness. They exhibited normal vision and were instructed to rest adequately the day before the experiment. Additionally, data regarding their educational background, specifically the number of years of schooling, were collected. Prior to scanning, all participants signed the informed consent form. The study protocol was approved by the Center for Cognition and Brain Disorders ethics committee at Hangzhou Normal University.

### Procedure

This study first collected resting-state fMRI (rs-fMRI) data from all participants in a quiet state. Subsequently, task fMRI data were acquired, during which participants judged the direction of a word picture shown on the screen (left or right). Following the procedure used in previous studies (Xue et al., 2023), word-selective regions were identified using a block-designed word localizer task. After completing the MRI data collection, participants performed two behavioral tasks involving visual form matching.

### fMRI data acquisition

We used a GE 3-Tesla MRI scanner (MR-750, GE Medical Systems, Milwaukee, WI) to acquire data at the Hangzhou Normal University Affiliated Hospital. Before entering the scanner, participants were instructed to remove metal objects from their bodies and lie flat on the examination bed. During the scan, they were asked to keep their heads as still as possible, and a sponge pad was used to minimize the head movement.

**Figure 1:**
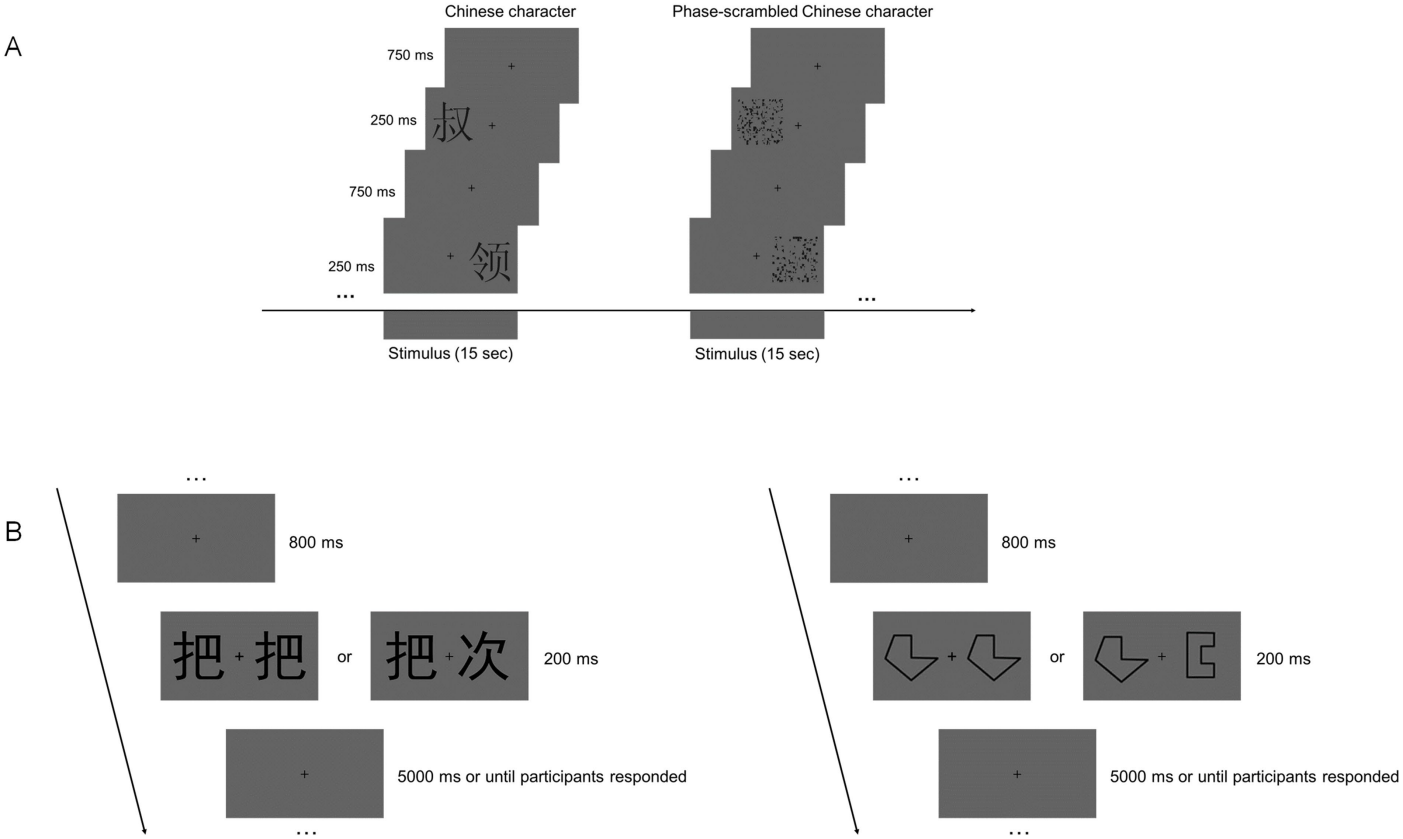
Design of the fMRI and behavioral experiments. **(A)** Example of stimulus blocks used in the localizer run. **(B)** Schematic depiction of the visual matching test in the behavioral experiment.

Echo Planar Imaging (EPI) was used to obtain rs-fMRI and task-fMRI data. The imaging parameters were as follows: 43 axial slices, repetition time (TR) = 2000 ms, time of echo (TE) = 30 ms, voxel size = 3.4 mm × 3.4 mm × 3.2 mm, flip angle (FA) = 90°, field of view (FOV) = 220 × 220 mm^2^, and matrix size = 64 × 64. The resting session comprised 240 contiguous volumes, lasting 8 min, followed by the task (localizer) session, which lasted 8 min and 10 s, resulting in a continuous time course of 245 time points. After acquiring the rs-fMRI data, participants performed a localizer task that involved a simple location judgment task. As shown in Figure 1A, visual stimuli were divided into two types: Chinese characters and phase-separated Chinese characters. The first fixation was presented for 10 s before the first stimulus appeared. The task included 16 blocks of visual stimulation and 16 blocks of fixation (“+”), each lasting 15 s in a balanced order. Each picture was presented for 250 ms, followed by a 750-ms inter-stimulus interval. Stimuli were presented on either the left or right side of the fixation point, and participants judged the location of the stimulus by pressing the corresponding button as accurately and quickly as possible.

### Behavioral data acquisition

After completing all MRI scans, participants rested before performing two visual matching tasks—word matching and symbol-form matching—in another room. This behavioral task aimed to investigate whether there is a specific decline in word perception among healthy older adults compared with young adults. The tasks were programmed and presented using E-Prime 2.0 and displayed on a 17-inch computer monitor (1,024 × 768 pixels at 60 Hz).

As shown in Figure 1B, the visual matching task includes 30 new pictures of words and 30 new pictures of symbols, all in stick figure style. In each trial, a fixed fixation point “+” is presented at the center of the screen for 800 ms. Then, two words appear for 200 ms, after which a gray blank screen is displayed. The trial proceeds to the next one after the participant presses a button. The maximum duration of each trial is 5,000 ms. The stimuli are black against the background, and the visual angle of each stimulus is 1.1. The test consists of 60 trials, with randomized stimuli and same/different trials. Participants must quickly determine whether the two words on the screen are identical and press the corresponding button as swiftly and accurately as possible. A baseline control test (symbol-form matching test) was used to control for general cognitive aging effects, following the same procedure as the word matching test. Due to procedural and recording issues, data from three participants in the young adult group were missing.

### Behavioral data analyses

Before further analysis, we excluded results with reaction times (RTs) longer than 3,000 ms or less than 200 ms (Qing et al., 2022; Xue et al., 2023). These trials comprised 0.008% of the total trials in the young adult group and 0.042% in the older adult group (*t* (36) = 4.140, *p* < 0.001).

Consistent with the procedures followed in previous studies (Qing et al., 2022; Xue et al., 2023) to reduce potential influence from the speed-accuracy trade-off, such as false effects of the experiment or the influences from different response strategies of the participant, and to clarify participants’ ability to process words and symbols, we used the inverse efficiency score (IES) as an indicator (Ashby and Gregory, 1978). It is calculated as the average correct reaction time (RT) divided by the proportion of correct responses (Vandierendonck, 2018).

### fMRI data analyses

We performed the following analyses to identify the between-group differences in functional connectivity within and between the word network and other networks. First, we processed localizer task-fMRI data and generated an activation map for word perception for each group (i.e., task-fMRI data processing). Subsequently, we created two regions of interest (ROIs) in the left and right vOT based on the activation map for each group (i.e., Regions of Interest Selection). Finally, we processed resting-state fMRI data and calculated seed-based functional connectivity within and between the word network and other networks using two ROIs for each group (i.e., Data Preprocessing for Resting-State fMRI Data and Seed-Based Functional Connectivity Analyses).

#### Task-fMRI data processing

The Data Processing and Analysis for Brain Imaging (DPABI) toolkit, based on MATLAB and developed by Yan et al. (2016), was used for task-fMRI data preprocessing. The preprocessing steps were identical to those in our previous study: removal of the first five time points, slice timing, realignment, spatial normalization to the EPI template in the Montreal Neurological Institute (MNI) space, and spatial smoothing using an isotropic Gaussian kernel with a full width at half maximum (FWHM) of 6 mm (Xue et al., 2023).

The general linear model (GLM) in SPM12 was used to generate activation maps (see Xue et al., 2023, for detailed information). The phase-scattered Chinese characters were used as a baseline in the localizer task, and the activation levels caused by the Chinese character images were compared. The linear contrast image (Chinese characters vs. scattered Chinese characters) was calculated to identify the brain regions sensitive to word perception for each participant. The one-sample *t*-test was performed to obtain the group-level activation map.

#### Regions of interest selection

Based on the activation levels obtained in previous studies (Cohen and Dehaene, 2004; He, 2015) and the results from the localizer task in the present study, we identified the left and right vOT regions as components of the word-selective areas. The vOT consisted of these areas in the Automated Anatomical Labeling (AAL) atlas, including Occipital_Sup, Occipital_Mid, Occipital_Inf, Fusiform, Lingual, Temporal_Sup, Temporal_Pole_Sup, Temporal_Mid, Temporal_Pole_Mid, Temporal_Inf, and ParaHippocampal (Tzourio-Mazoyer et al., 2002; Price and Devlin 2011). Specifically, for each group, the most activated voxels in the activation map (FDR correction, *q* < 0.05) within the left and right vOT of each region were selected as the seed voxels (see Table 1 for details).

**Table 1:** Seeds selected from the group-level activation map during the word localizer task in older adults (left) and young adults (right).

ROIs	Older adults				Young adults			
	MNI coordinates			Peak *t* value	MNI coordinates			Peak *t* value
	X	Y	Z		X	Y	Z	
lvOT	−42	−54	−21	8.102	−39	−45	−15	7.173
rvOT	45	−48	−24	6.998	45	−75	−15	6.834

Two spherical regions of interest (ROIs), each with a radius of 5 mm and centered on the two seed voxels, were then created for each group (see Supplementary Figure S1 for detailed information).

#### Data preprocessing for resting-state fMRI data

Resting-state fMRI data were preprocessed using the DPABI toolkit, following the same procedure as our previous study: removal of the first 10 time points, slice timing correction, realignment, spatial normalization to the EPI template in MNI space, spatial smoothing (FWHM = 6 mm), removal of the linear trend, regression of covariates including head motion profiles (Friston 24-parameter, Friston et al., 1996), white matter and cerebrospinal fluid signals, and band-pass filtering (0.01–0.08 Hz) (Qing et al., 2022).

#### Seed-based functional connectivity analyses

Within the word network, we conducted FC analyses. For each participant, the Pearson correlation coefficient was calculated between the averaged time courses of two ROIs, i.e., left and right vOT. The correlation coefficients were subsequently transformed into z-values via Fisher’s r-to-z transformation.

Beyond the word network, we conducted FC analyses. For each participant, the Pearson correlation coefficients were computed between the averaged time course of each ROI and the time course of every voxel in the brain. The resulting correlation coefficients were then transformed to *z*-values using Fisher’s r-to-z transformation.

### Statistical analyses

Behavioral data of the visual-form matching task were analyzed using a 2 × 2 repeated-measure analysis of variance (ANOVA). The between-subject variable was age (older adults vs. young adults), and the within-subject variable was stimulus type (word pictures vs. symbol pictures). To further investigate whether there was a specific decline in word perception ability among older adults, we conducted an independent-sample t-test to compare the difference in IES between word and symbol processing. These correlation analyses were conducted with a threshold *p-value of* < 0.05.

To assess between-group differences in FC within the word network, we conducted a two-sample *t*-test comparing the connectivity values of the two vOT ROIs between older and young adult groups. The resultant *t*-value was thresholded with a *p-*value of < 0.05. For FC beyond the word network, two-sample *t*-tests were performed within the Automated Anatomical Labeling (AAL) mask (Tzourio-Mazoyer et al., 2002), with gender included as a covariate. Gaussian Random Field (GRF) theory was applied for multiple comparison correction, with a *p-*value of <0.001 for voxel-level and a *p-*value of <0.05 for cluster-level significance (two-tailed).

Finally, to examine the relationship between the connectivity changes and behavioral performance, a Pearson correlation coefficient was calculated between the mean connectivity value of each cluster showing significant between-group differences and the IES from the word-matching task. Moreover, to minimize the impact of general aging on the results, we computed partial correlations between the connectivity value in each cluster and the IES from the word-matching task, controlling for the IES from the symbol-form matching task (calculated using the ppcor R package; Kim, 2015). These correlation analyses were thresholded with *p* < 0.05.

## Results

### Demographic characteristics

The average age of participants in the older adult group was significantly higher than in the young adult group (*t* (23.08) = 40.546, *p* < 0.001). Both groups had completed their junior high school or higher education. All participants completed the Combined Raven’s Test (CRT) in its Chinese version, published in 1991 (Li and Chen, 1989). This standardized assessment is extensively utilized for evaluating non-verbal intelligence quotient (IQ) and indicates general cognitive ability (Zhao et al., 2019; Xue et al., 2023; Xue et al., 2020). There was no significant difference in scores of the Raven test between the older and young adult groups (*t* (25.84) = −1.548, *p* = 0.134). Additionally, there was no significant difference in gender distribution (*χ^2^* = 0.256, *p* = 0.613). The number of years of schooling for the older adults (mean = 12.60 years, SD = 2.74) was significantly less than that of young adults (mean = 15.89 years, SD = 1.54) (*t* (27.38) = −4.64, *p* < 0.001).

### Between-group difference of behavioral performances in visual matching task

**Figure 2:**
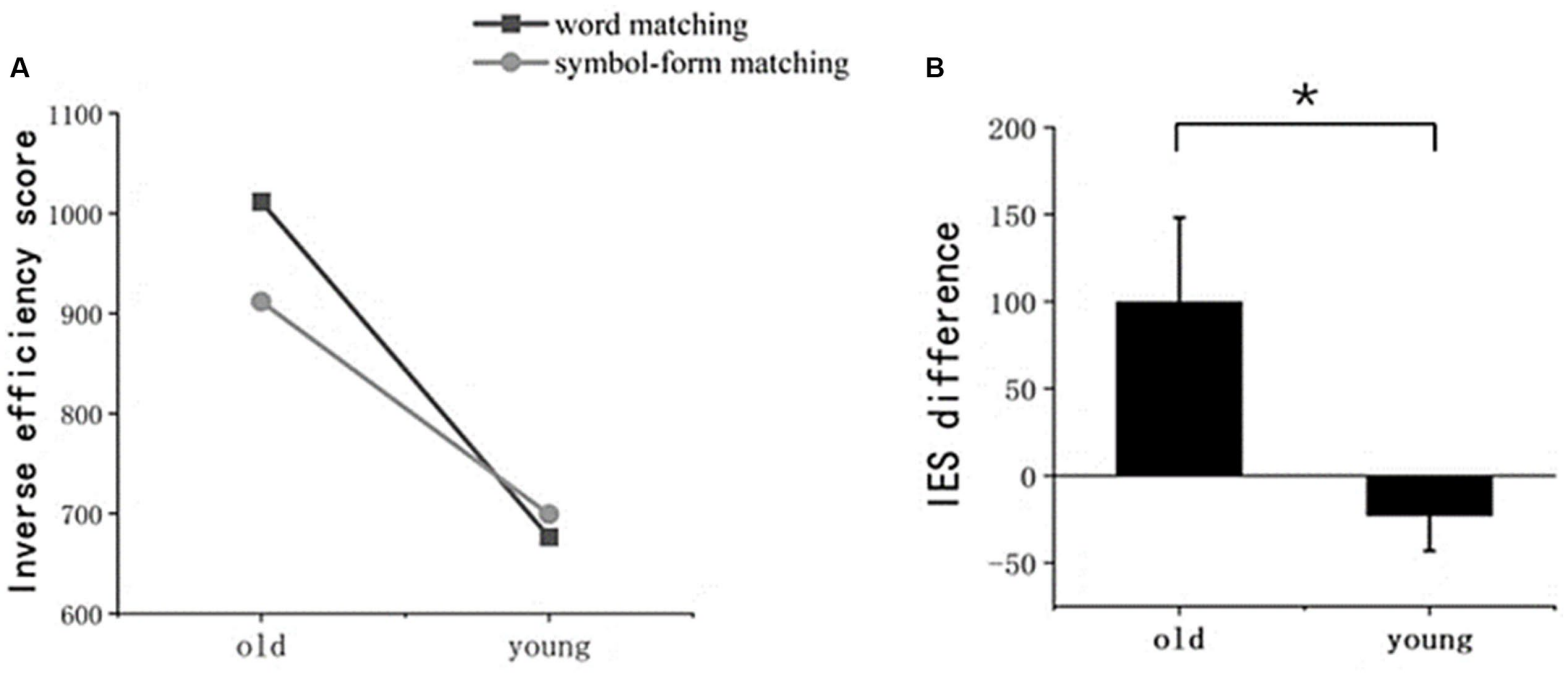
Performance in the behavioral experiment. **(A)** Inverse efficiency scores (IES) for word and symbol-form matching tests in both older and young adult groups. **(B)** The difference in IES between the word and symbol-form matching tests for these two groups. The error bar denotes the SD.

Figure 2 illustrates the inverse efficiency scores (IES) for young and older adults. A 2 × 2 repeated measures ANOVA revealed a significant main effect of age group (*F* (1, 36) = 50.842, *p* < 0.001), indicating that older adults had significantly higher IES compared to the young adults in both word- and symbol-matching tasks. Additionally, there was a significant interaction between the stimulus type and age group (*F* (1, 36) = 5.456, *p* = 0.025), showing that the decline in performance for word processing was more pronounced in older adults. Furthermore, only older adults exhibited significantly worse performance (i.e., higher IES) in the word-matching task compared to the symbol-matching task (*p* = 0.011).

To reduce the influence of general aging on the results, we used the IES difference between word matching and symbol matching as a new indicator. The independent-sample *t*-test revealed that the IES difference was significantly greater in older adults than in young adults (*t* (24.149) = 2.336, *p* = 0.028).

### Between-group difference in resting-state functional connectivity

First, we found that older adults exhibited increased FC between the two word-selective ROIs (i.e., the left vOT to the right vOT, within the word network) compared to young adults (*t* (39) = 5.133, *p* < 0.001).

**Figure 3:**
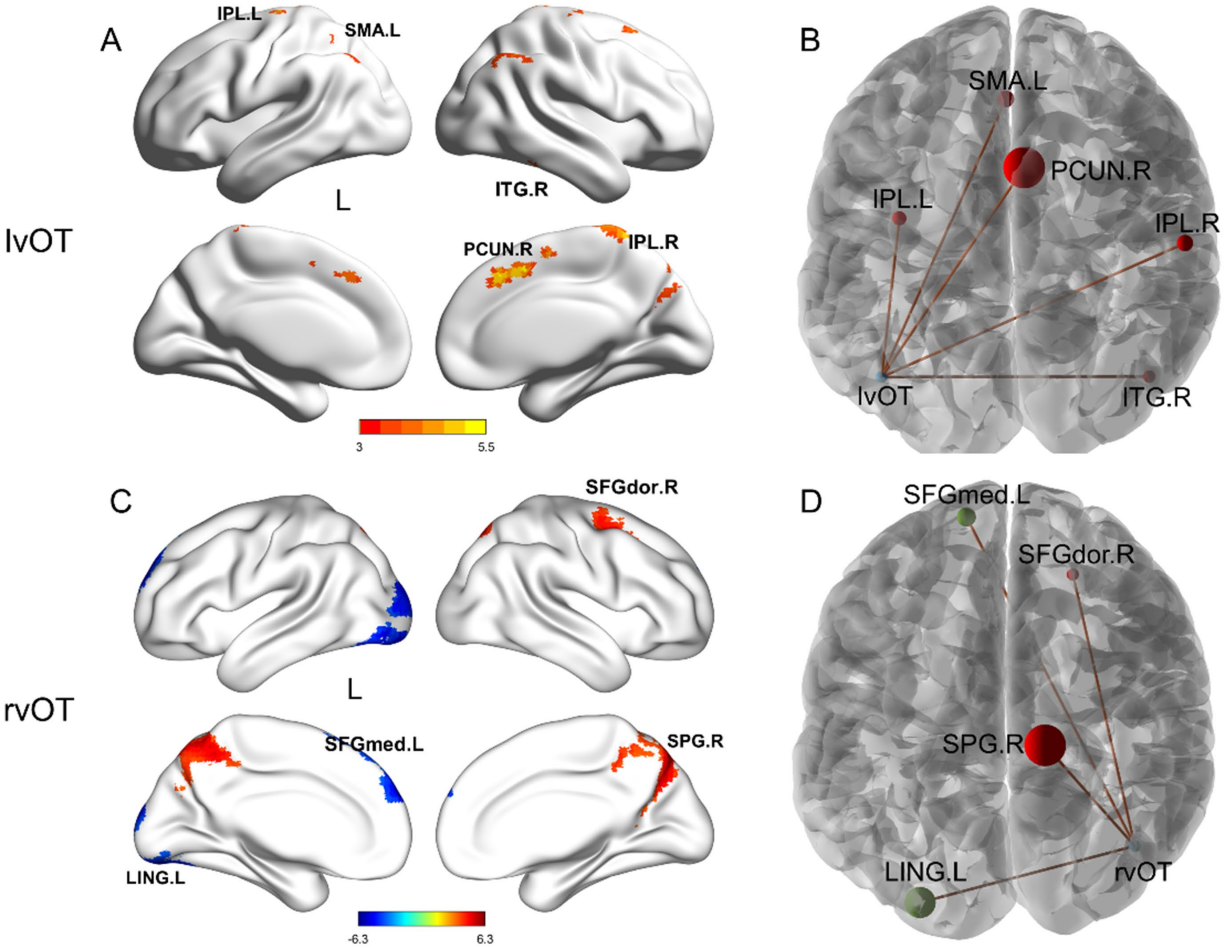
Between-group differences in seed-based FC beyond the word network. Compared to the young adults, older adults showed **(A,B)** increased FC between the lvOT and other brain areas, including IPL.L, SMA.L, PCUN.R, IPL.R, and ITG.R; **(C,D)** increased FC between the rvOT and SPG.R, SFGdor.R; and decreased FC between the rvOT and LING.L, and SFGmed.L. The left panel depicts the regions exhibiting differential activation along with the intensity of these differences. In the right panel, the size of the spheres represents the cluster size of FC difference, with red indicating higher connectivity strength in older adults compared to young adults. r, right hemisphere; l, left hemisphere; vOT, ventral occipitotemporal area; ROI, region of interest. Brain regions were visualized using the BrainNet viewer (Xia et al., 2013).

Second, the results of between-group differences in connectivity between the two word-selective ROIs and other non-word-selective regions (i.e., beyond the word network) are summarized in Table 2 and illustrated in Figure 3. Older adults exhibited increased FC beyond the word network than young adults. Specifically, FC between the left vOT and the right inferior temporal gyrus (ITG.R), right inferior parietal (IPL.R), left inferior parietal (IPL.L), supplementary motor area (SMA.L), and right precuneus (PCUN.R) was found to be increased in older adults. Similarly, FC between the right vOT and the right superior parietal gyrus (SPG.R) and right dorsolateral superior frontal gyrus (SFGdor.R) also increased in older adults compared to young adults. Conversely, FC between the right vOT and the left lingual gyrus (LING.L) and left medial superior frontal gyrus (SFGmed.L) decreased in older adults compared to young adults. Furthermore, when controlling for the number of years of education as a covariate, the between-group differences in FC remained consistent (see Supplementary Table S1 for detailed information).

**Table 2:** Regions exhibited significant between-group differences in FC beyond the word network.

ROIs	Regions	Cluster size	Peak *t* value	MNI coordinates		
				X	Y	Z
lvOT	ITG.R	88	5.327	48	–51	–24
	IPL.R	108	4.337	60	–54	42
	IPL.L	66	4.644	−36	–54	54
	SMA.L	149	5.293	0	6	51
	PCUN.R	470	5.047	6	–45	69
rvOT	SPG.R	733	6.287	15	–78	54
	LING.L	511	−6.290	−27	–84	–15
	SFGmed.L	254	−5.792	−12	57	27
	SFGdor.R	106	4.972	24	3	54

*ITG.R = right inferior temporal gyrus, IPL.R = right inferior parietal, but supramarginal and angular gyri, IPL.L = left inferior parietal, but supramarginal and angular gyri, SMA.L = left supplementary motor area, PCUN.R = right precuneus, SPG.R = right superior parietal gyrus, LING.L = left lingual gyrus, SFGmed.L = left superior frontal gyrus, medial, SFGdor.R = right superior frontal gyrus, dorsolateral.

### Brian-behavior relationship

**Figure 4:**
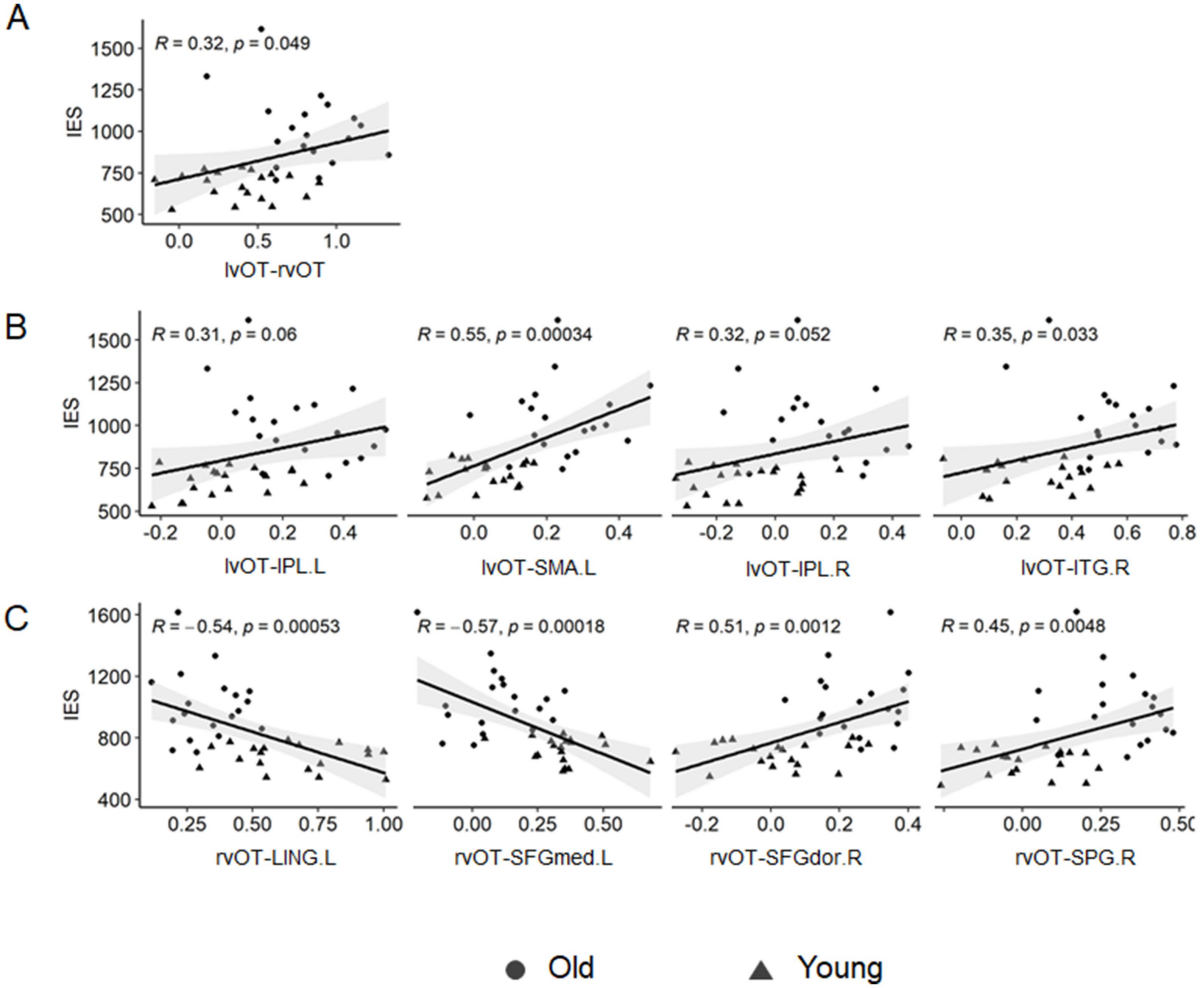
Correlation between seed-based FC and visual word matching performance. **(A)** The lvOT-rvOT FC was positively correlated with the IES of a visual word-matching task. **(B)** The lvOT-non-word-selective regions FC was also positively correlated with the IES of a visual word-matching task. **(C)** The rvOT-non-word-selective regions (SFGdor.R and SPG.R) FC was positively correlated with the IES of the visual word-matching task, whereas the rvOT-LING.L FC and the rvOT-SFGmed.L FC were negatively correlated with the IES of the visual word-matching task.

We conducted Pearson correlation analyses to explore the relationship between connectivity changes and behavioral performance. The correlation results are presented in Table 3 and Figure 4.

**Table 3:** Correlation between FC and word-matching task performance.

ROIs	Regions	WMT_*r*	WMT_ *p*	WMT par_*r*	WMT par_ *p*
lvOT	rvOT	0.32	0.049^*^	−0.03	0.860
lvOT	ITG.R	0.35	0.033^*^	−0.05	0.764
	IPL.R	0.32	0.052^#^	0.01	0.967
	IPL.L	0.31	0.006^*^	−0.07	0.691
	SMA.L	0.55	0.001^*^	0.31	0.058^#^
	PCUN.R	0.31	0.062^#^	−0.12	0.476
rvOT	SPG.R	0.45	0.005^*^	0.17	0.315
	LING.L	−0.54	0.001^*^	−0.27	0.095^#^
	SFGmed.L	−0.57	0.001^*^	−0.42	0.010^*^
	SFGdor.R	0.51	0.001^*^	0.27	0.101

WMT, IES for the word-matching task; FMT, IES for the symbol-form matching task; *r*, correlation coefficient; *p*, significance; par, partial correlation, controlling IES of FMT; * *p* < 0.05, # *p* < 0.01.

As shown in Table 3 and Figure 4, the IES of the word-matching task was significantly or marginally significantly correlated with the lvOT-rvOT connectivity (*r* = 0.32, *p* = 0.049), lvOT-ITG.R connectivity (*r* = 0.35, *p* = 0.033), lvOT-IPL.R connectivity (*r* = 0.32, *p* = 0.052), lvOT-IPL.L connectivity (*r* = 0.31, *p* = 0.006), lvOT-SMA.L connectivity (*r* = 0.55, *p* = 0.001), and lvOT-PCUN.R connectivity (*r* = 0.31, *p* = 0.062). Similarly, the IES of the word-matching task was positively correlated with the rvOT-SPG.R connectivity (*r* = 0.45, *p* = 0.005) and rvOT-SFGdor.R connectivity (*r* = 0.51, *p* = 0.001). Conversely, the IES of the word-matching task was negatively correlated with rvOT-LING.L connectivity (*r* = −0.54, *p* = 0.001) and rvOT-SFGmed.L connectivity (*r* = −0.51, *p* = 0.001). Importantly, partial correlation analyses further revealed that the lvOT- SMA.L FC (*r* = 0.31, *p* = 0.058) and the rvOT- SFGmed.L FC (*r* = −0.42, *p* = 0.010) were significantly or marginally significantly correlated with the IES of the word-matching task, after controlling for the influence of the IES of the symbol-form matching task.

## Discussion

The primary objective of this study was to investigate the intrinsic brain network characteristics of visual word perception in older adults. Our results indicated that resting-state functional connectivity (FC) between the two word-selective regions and between these regions and non-word-selective regions was found to be increased in older adults compared to young adults. Additionally, these age-related changes in FC were associated with cognitive decline in visual word processing among older adults. Taken together, these findings suggest that impaired word processing is linked to decreased segregation within and beyond the word network in the aging brain.

Consistent with the previous results (Gollan and Brown, 2006; Su et al., 2017; Xue et al., 2023), our results indicated that older adults had more difficulty processing words than young adults, suggesting age-related changes in visual word processing. Specifically, older adults performed worse than young adults in the word form-matching task. Furthermore, we observed an interaction between the stimulus type and age group, revealing a greater difference in performance between word and symbol-form matching in older adults compared to young adults. This finding suggests that older adults experience a more pronounced decline in word processing compared to symbol-form processing.

The first significant finding is decreased segregation within and beyond the word network in the aging brain. Specifically, we observed increased resting-state FC between the two word-selective regions (i.e., the left vOT and the right vOT) in older adults compared to young adults. Similarly, FC between the left vOT and other non-word-selective regions (i.e., ITG.R, IPL, SMA.L, and PCUN.R) was also found to be increased in the older adults compared to young adults. Previous task-based fMRI studies have shown that the left vOT exhibits stronger neural responses to visual words compared to other visual stimuli in young adults (Cohen et al., 2002; Cohen and Dehaene, 2004). In contrast, older adults demonstrate decreased selectivity for words in the left vOT (Park et al., 2004; Xue et al., 2023). These findings suggest neural dedifferentiation in the left vOT in the aging brain. Our results, indicating decreased segregation between the left vOT and other non-word-selective brain regions, support the neural dedifferentiation hypothesis from an intrinsic brain network perspective. Broadly, this study extends previous research by suggesting that decreased selectivity for words in the left vOT may be associated with increased FC between the left vOT and other regions in the aging brain (Xue et al., 2023). Our findings also extend the influential interactive specialization theory, which posits that the development of word selectivity in the left vOT involves not only changes in neural activity within this specific region but also changes in interactions between this region and other brain areas (see Johnson, 2000, 2001, 2005, for reviews).

A fundamental question arises regarding why older adults exhibit broad neural dedifferentiation in the intrinsic brain network, characterized by decreased segregation within and beyond the word network. One possible explanation is that the right vOT and other non-word-selective regions may compensate for the reduced processing efficiency of specific word-selective regions, such as the left vOT (Cohen et al., 2002; Cohen and Dehaene, 2004; Tarkiainen et al., 1999). Consistent with this notion, previous studies have found reduced selectivity of neural responses to words in the left vOT in older adults compared to young adults, indicating diminished processing efficiency in word-selective regions (Park et al., 2004; Xue et al., 2023). Additionally, non-word-selective regions are involved in various cognitive functions. For instance, the precuneus, inferior temporal gyrus, and angular gyrus are key hubs in the default network, which are involved in episodic memory and other functions (see Seghier, 2013, for further discussion). The putamen, part of the executive-control network, is involved in sustained attention (Seeley et al., 2007) and decision-making (Kuo and Nitsche, 2015). Thus, it is possible that increased FC between word-selective regions and non-word-selective regions reflects a compensatory mechanism. Moreover, previous studies have observed broadened connectivity in the aging brain between regions belonging to different networks, such as increased connectivity between the default network and the frontoparietal network (Grady et al., 2016; Huang et al., 2015), the default network and the auditory network (Huang et al., 2015), and the somatomotor network and visual network (Geerligs et al., 2015). However, further research is needed to verify these explanations.

The second key finding is that changes in resting-state FC are related to cognitive decline in visual word matching. Specifically, our results indicate that stronger FC between the two word-selective regions is associated with poorer word-processing performance. FC between word-selective and non-word-selective regions also correlates with word processing performance. Furthermore, even after accounting for general aging effects, the FC between the left vOT and the supplementary motor area (lvOT-SMA.L) and between the right vOT and the medial superior frontal gyrus (rvOT-SFGmed.L) remained correlated with word-matching task performance. This suggests a specific link between changes in brain connectivity and decline in visual word processing.

Similarly, previous studies have shown that changes in FC in various brain regions are closely associated with cognitive performance decline in aging (e.g., face processing, as noted by Qing et al., 2022, see also Ferreira and Busatto, 2013, for a review). Our study supports the relationship between behavior and resting-state FC, providing converging evidence that this relationship is prevalent across different cognitive functions affected by aging. Our findings suggest that decreased segregation within and beyond the word network in the brain may contribute to age-related decline in visual word processing. In a broader context, our results offer support for the neural dedifferentiation hypothesis regarding cognitive decline in visual word processing among older adults.

Notably, there appears to be a discrepancy between our findings and those of previous studies. He (2015) reported that skilled readers showed enhanced functional connectivity (FC) between the left ventral occipital temporal (vOT) region and several language-related brain areas, such as Broca’s area, when compared to illiterate individuals. At the individual level, FC between the left vOT and these language-related areas was positively associated with visual word reading proficiency (He, 2015; Zhang et al., 2014). In contrast, our study observed increased FC among regions in older adults with poorer word-processing abilities. This discrepancy might be interpretable from a developmental standpoint. According to the existing literature, the developmental trajectory is not linear but follows an inverted U-shaped pattern (Maurer et al. (2006), provides a detailed discussion). As individuals transition from pre-reading children to skilled readers (young adults), graphemes become linked with phonetics and semantics, and the connectivity between the left vOT and language-related brain areas is enhanced, ultimately leading to the formation of a specialized reading neural network.

Consequently, FC between the left vOT and language-related brain areas correlates positively with visual word reading performance in children and young adults (He, 2015; Zhang et al., 2014). However, as individuals progress from young adulthood to older adulthood, a decline in visual word perception is observed behaviorally, and there is a reduction in the neural selectivity for words in the left vOT (i.e., neural dedifferentiation) (Xue et al., 2023; Park et al., 2004). The efficiency of the left vOT diminishes, and it may require increased interaction with language-related areas to compensate. This could explain the increased FC in older adults compared to young adults in our study. Nevertheless, this remains a significant and unresolved question that warrants further research.

More broadly, our results enhance the understanding of the intrinsic brain network changes associated with declined processing of perceptual expert stimuli in older adults. Recently, Qing et al. (2022) examined the resting-state FC related to aging in face processing and found that, compared to young adults, older adults exhibited decreased FC between face-selective regions (e.g., the fusiform face area and the occipital face area) but increased FC between these face-selective regions and non-face-selective regions. Similarly, the present study observed increased FC between word-selective and between word-selective and non-word-selective regions in older adults. These findings, combined with those from previous studies, suggest a universal change in the intrinsic brain network underlying the declined processing of various expert stimuli, characterized by decreased segregation beyond the specific stimuli-selective network in the aging brain. However, differences may exist in the interaction between brain regions within specific stimuli-selective networks, such as decreased integration within the face-selective network and decreased segregation within the word-selective network. The mechanisms underlying these changes in segregation and integration require further investigation.

Collectively, our study contributes to the understanding of the neural substrates of aging in visual word perception. First, our findings from resting-state brain networks offer novel empirical support for theories concerning the perceptual decline in visual word processing, specifically the neural dedifferentiation hypothesis. Second, we demonstrated that alterations in resting-state functional connectivity (FC) are closely associated with behavioral performance in word-matching tasks. These findings suggest a potential neural biomarker for cognitive aging in word perception, potentially illuminating new avenues for assessing cognitive decline in healthy older adults. Third, our observations of older adults provide insights into the development of the brain network for word processing at later life stages and contribute new empirical evidence to the interactive neural specialization theory.

Before concluding the discussion, we acknowledge two limitations. First, while general cognitive ability, as reflected in IQ scores, was matched between older and young adults, it remains challenging to completely exclude potential effects related to differences in specific cognitive abilities, such as memory. There may also be other underlying factors, such as cognitive visual skills, that were not directly measured in our study. Future research should incorporate a more comprehensive set of cognitive assessments to mitigate the impact of these variables and provide a clearer interpretation of the results. Second, although the visual word-matching task primarily assesses the perceptual processing of visual word forms, phonological and semantic processing may also be engaged automatically. Future studies should incorporate multiple tests to better assess the level at which aging affects visual word processing. Third, the sample size in our study is limited. Therefore, it is imperative for future studies to validate our findings using a larger participant pool. Additionally, while the white matter is considered a crucial component of brain networks, and research has shown that BOLD signals within the white matter are instrumental for understanding both normal cognitive functions and neurological conditions (Ji et al., 2017; Ji et al., 2023), our study did not directly assess white matter functionality. This omission represents a significant area for future exploration and research.

## Conclusion

Age-related changes in intrinsic connectivity may reflect the neural correlates of cognitive aging in visual word processing. Specifically, we observed decreased segregation within and beyond the word network in the aging brain. Additionally, these changes in functional connectivity were closely associated with a decline in word processing performance. These findings support the neural dedifferentiation hypothesis of age-related decline in word processing from an intrinsic brain network perspective.

## Data Availability

The raw data supporting the conclusions of this article will be made available by the authors, without undue reservation.
