# Supplementary material for: Aging of visual word perception is related to decreased segregation within and beyond the word network in the brain
Source: Front Aging Neurosci. 2024 Dec 5;16:1483449. doi: 10.3389/fnagi.2024.1483449 (PMC11655501; doi:10.3389/fnagi.2024.1483449)
Supplement: Supplementary file 1 [file Data_Sheet_1.docx]

**Supplementary Materials**

**
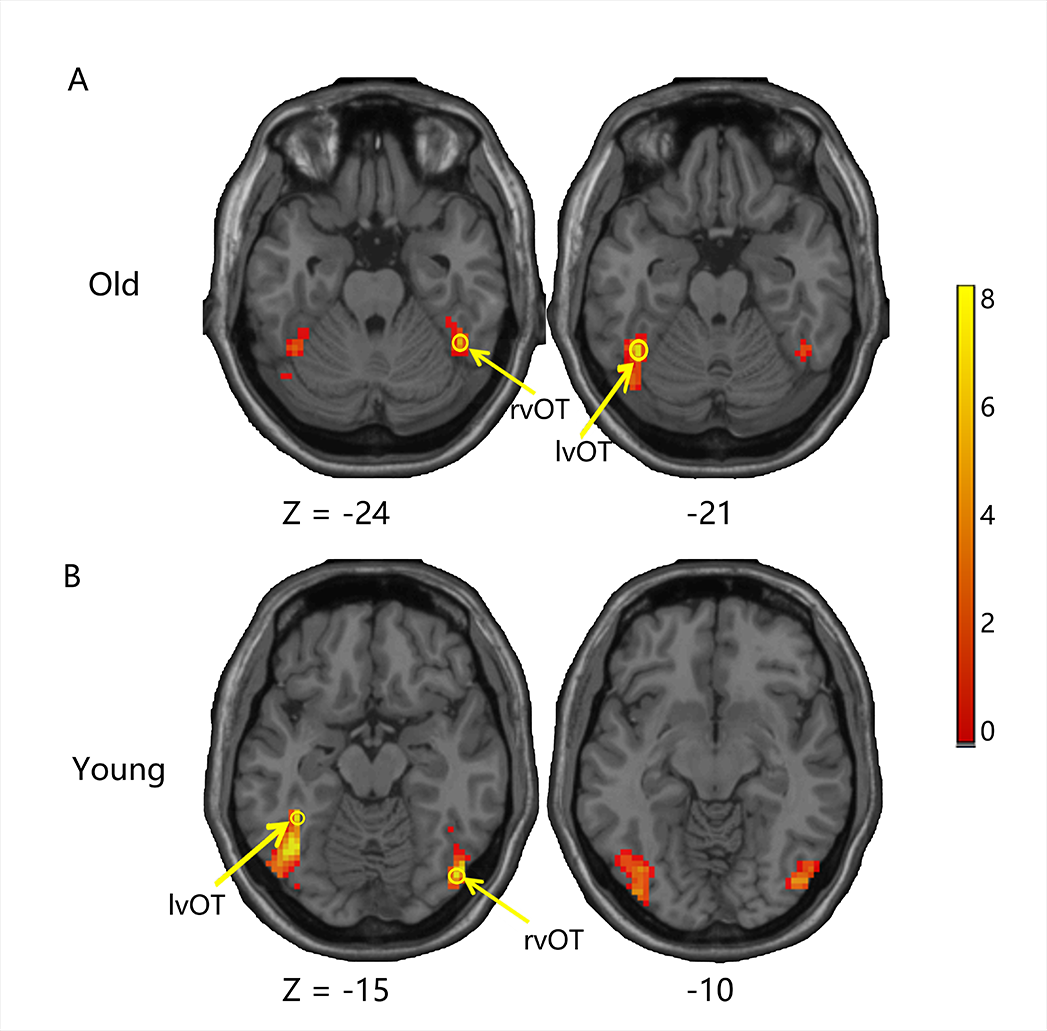
**

**Supplementary Figure 1. ROI selection.** Selection of regions sensitive to word perception in the left and right vOT based on group-level activation map during the localizer task in (A) old adults and (B) young adults (FDR correction, *q* < 0.05). Yellow circles indicated the left and right vOT seeds; r: right hemisphere; l: left hemisphere; vOT: ventral occipitotemporal area.

**Supplementary Table 1** Group comparisons results for regions exhibited significant between-group differences in FC using the years of schooling as a covariate

| **ROIs** | **Regions** | ***t*-value** | ***p-value*** |
| --- | --- | --- | --- |
| **lvOT** | **rvOT** | 3.119 | 0.003 |
| **lvOT** | ITG.R | 3.644 | 0.001 |
|  | IPL.R | 2.663 | 0.011 |
|  | IPL.L | 3.152 | 0.003 |
|  | SMA.L | 3.457 | 0.001 |
|  | PCUN.R | 3.763 | 0.001 |
| **rvOT** | SPG.R | 3.676 | 0.001 |
|  | LING.L | -2.737 | 0.009 |
|  | SFGmed.L | -2.724 | 0.010 |
|  | SFGdor.R | 2.632 | 0.012 |

Notes: vOT: ventral occipitotemporal area; r: right hemisphere; l: left hemisphere; ITG.R = right inferior temporal gyrus, IPL.R = right inferior parietal , but supramarginal and angular gyri, IPL.L = left inferior parietal, but supramarginal and angular gyri, SMA.L = left supplementary motor area, PCUN.R=right precuneus, SPG.R=right superior parietal gyrus, LING.L= left lingual gyrus, SFGmed.L=left superior frontal gyrus, medial, SFGdor.R = right superior frontal gyrus, dorsolateral.
